# A multi-institutional and cross-sectional study on empathy in Chinese medical students: differences by student cadre or not, future career preference, and father’s education status

**DOI:** 10.1186/s12909-020-1935-x

**Published:** 2020-01-28

**Authors:** Kangqi Yi, Mingyi Kang, Dongju Li, Zhipeng Wang, Jinsong Bai, Huiming Xu, Shulan Ma

**Affiliations:** 1 School of Public Health, Fudan University, Shanghai, China; 2 Experimental Teaching Center of Basic Medical Science, School of Basic Medical Sciences, Fudan University, Shanghai, China; 3 School of Social Development and Public Policy, Fudan University, Shanghai, China; 4 Shimen Second Road Community Health Service Center, Shanghai, China; 5 Medical Education and Medical Humanities Research Center, School of Basic Medical Sciences, Fudan University, P.O. Box 192, 138 Yi-Xue-Yuan Road, Shanghai, 200032 China

**Keywords:** Empathy, China, Jefferson scale of empathy-student version, Medical students, Undergraduate

## Abstract

**Background:**

The aims of this study were to examine the factor structure of the Chinese version of the Jefferson Scale of Empathy for medical students (JSE-S) and investigate differences in empathy scores among Chinese medical students according to gender, student cadre or not, future career preference, and parents’ education.

**Methods:**

Medical students from three universities completed an online questionnaire containing the JSE-S. Exploratory factor analysis was conducted to determine the factor structure, and group comparisons of empathy scores were examined via *t*-tests and analysis of variance.

**Results:**

Four factors emerged from the factor analysis: “perspective taking,” “compassionate care,” “standing in the patient’s shoes,” and an uninterpretable factor. The results indicated that students who were female, held positions as student cadres, preferred to become a doctor, and whose fathers had a high school education or below tended to have more empathy.

**Conclusions:**

Overall, the findings provide information on the dimensions of empathy applicable to Chinese medical students and confirm the factors found in the original measure. The dimensions have implications for developing empathy among medical students throughout the world. Educators can use the information to design interventions to foster empathy among students in the context of medical education reform in many countries, including China.

## Background

Empathy in patient care was defined by Hojat et al. [1] as a predominantly cognitive characteristic that involves not only an understanding of patients’ experiences, concerns, and perspectives but also a capacity to communicate this understanding to patients and an intention to help them. Based on a comprehensive review of relevant literature, it is evident that empathy plays an essential role in achieving optimal outcomes in medical care by optimizing careful history taking, diagnostic accuracy, compliance to treatment, and maximum utilization of resources, thereby increasing patients’ satisfaction and quality of health care [2–4]. As such, it would be fruitful for medical school faculty to promote the cultivation of empathy among medical students as part of the contemporary “professionalism” system in medicine [5]. The value of empathy in the practice of medicine has attracted much attention among medical educators, rendering the measurement and evaluation of empathic ability an important area of research investigation.

The Jefferson Scale of Empathy (JSE), developed by Hojat and his colleagues at Jefferson Medical College, was designed specifically in the context of medical care [6]. Multiple studies have used the JSE-S to assess empathy in medical students in a variety of cultural and linguistic settings, thereby generating comparable results from different countries. However, the research regarding Chinese students is very limited. Consequently, this study investigated empathy in medical students at three comprehensive universities in Shanghai, China. As such, this is the first multi-institutional study of medical students in China, allowing for a diversity of participants in the study. First, we examined the factor structure of the Chinese version of the JSE-S. Then, we assessed differences in empathy scores by certain characteristics including gender, student cadre, future career preference, and parents’ level of education.

## Methods

### Participants

We adopted a multi-institutional, cross-sectional design. Participants included medical students from Fudan University, Shanghai Jiaotong University, and Tongji University who majored in clinical medicine in a five- or eight-year program. In total, 528 medical students participated in the study. We then conducted some logical checks on the questionnaire results and excluded seven questionnaires due to inconsistent data, resulting in a final sample of 521 participants.

### Measure

The self-assessed questionnaire comprised two sections: Section A focused on the demographic characteristics, such as students’ gender and age. Section B was the Chinese version of the JSE-S [7] used to assess empathy. The measure consists of 20 items that are responded to on a 7-point Likert-type scale (1 = *strongly disagree*, 7 = *strongly agree*). Half the items are positively worded, and half are negatively worded. Total scores range from 20 to 140. Higher scores indicate a higher degree of empathy. The psychometric properties of this scale have been described as satisfactory [7].

### Procedures

We approached the student union in three medical schools of comprehensive universities in August 2018 and recruited no-interest-related student cadres as investigators. After a standard training was conducted for investigators to foster a common understanding of the research, they posted the online questionnaire and invited students to participate voluntarily during the same period in the three universities. We provided an explanatory statement to the potential participants and informed them that the results would be reported only in aggregate for research purposes, ensuring their anonymity and confidentiality. Completion of the questionnaire indicated their consent. The study protocol was approved by the Fudan University Research Ethics Committee.

### Statistical analyses

Pearson correlation coefficients were calculated to examine item-total score correlations. After a Kaiser-Meyer-Olkin measure of sampling adequacy and Bartlett’s test of sphericity, exploratory factor analysis (principal component factor extraction) with varimax rotation was employed to examine the underlying factor structure. Cronbach’s alpha was calculated to assess internal consistency reliability. Differences in gender, student cadre or not, and parents’ education status were evaluated using *t*-tests. Analysis of variance, including post hoc tests, was used to compare empathy scores among students with different future preferences. We performed statistical analysis using SPSS 22.0 (IBM, Armonk, NY, USA), with the significance level set at .05.

## Results

### Descriptive statistics

Approximately 11.45% of all targeted students at the three universities effectively completed the questionnaire. Table 1 describes the characteristics of the 521 participants. The majority of the participants were female students (64.88%) and aged 20–22 years (57.97%). Most were students from Jiaotong University (37.62%), followed by Fudan University (33.78%), and Tongji University (28.60%).

**Table 1:** Participant’s demographic information

Variables	Fudan Universtiy	Jiaotong University	Tongji University	Total
Total	176 (33.78%)	196 (37.62%)	149 (28.60%)	521 (100.00%)
Gender				
Male	67 (12.86%)	62 (11.90%)	54 (10.36%)	183 (35.12%)
Female	109 (20.92%)	134 (25.72%)	95 (18.23%)	338 (64.88%)
Age (years)				
< 20	51 (9.79%)	73 (14.01%)	62 (11.90%)	186 (35.70%)
20–22	120 (23.03%)	108 (20.73%)	74 (14.20%)	302 (57.97%)
23–25	4 (0.77%)	15 (2.88%)	10 (1.92%)	29 (5.57%)
> 25	1 (0.19%)	0 (0.00%)	3 (0.58%)	4 (0.77%)
Medical school year				
1st year	29 (5.57%)	46 (8.83%)	39 (7.49%)	114 (21.88%)
2nd year	47 (9.02%)	82 (15.74%)	62 (11.90%)	191 (36.66%)
3rd year	62 (11.90%)	28 (5.37%)	36 (6.91%)	126 (24.18%)
4th year	32 (6.14%)	32 (6.14%)	9 (1.73%)	73 (14.01%)
5th year and above	6 (1.15%)	8 (1.54%)	3 (0.58%)	17 (3.26%)

Means, standard deviations, medians, and score ranges for the three universities and for all students are provided in Table 2. The total mean was 104.66 (*SD* = 13.09), and scores ranged from 39 to 137. Most items produced a mean score higher than 5 (see Table 3). Cronbach’s alpha in our study was 0.82.

**Table 2:** Descriptive statistics for scores on the JSE-S

Parameters	Fudan Universtiy	Jiaotong University	Tongji University	Total
Mean ± SD	103.59 ± 13.53	105.17 ± 12.21	105.24 ± 13.67	104.66 ± 13.09
Median(50% percentile)	105.00	106.00	106.00	106.00
25% percentile	96.00	97.00	96.50	97.00
75% percentile	113.00	114.00	114.00	114.00
Possible range	20–140	20–140	20–140	20–140
Actural range	39–132	68–134	76–137	39–137
Cronbach’s α-coefficient				0.82

**Table 3:** Rotated factor solution, item means and standard deviations, and item-total score correlations

Item^a^	Factor 1	Factor 2	Factor 3	Factor 4	Mean/ SD	r_it_
Physicians’ understanding of the emotional status of their patients, as well as that of their families, is one important component of the physician–patient relationship. (16)	**.797**	.268	−.044	.017	5.71/1.15	.70^**^
I believe that empathy is an important therapeutic factor in the medical treatment. (20)	**.782**	.144	.060	.051	5.73/1.21	.65^**^
Physicians should try to think like their patients in order to render better care. (17)	**.762**	.047	.042	.080	5.30/1.25	.58^**^
Physicians should try to understand what is going on in their patients’ minds by paying attention to their nonverbal cues and body language. (13)	**.726**	.050	.024	.012	5.35/1.29	.55^**^
Physicians should try to stand in their patients’ shoes when providing care to them. (9)	**.722**	.162	−.056	−.048	5.69/1.19	.59^**^
Patients value a physician’s understanding of their feelings which is therapeutic in its own right. (10)	**.705**	.265	−.103	−.052	5.85/1.15	.62^**^
Empathy is a therapeutic skill without which the physician’s success is limited. (15)	**.690**	−.093	.161	.165	5.91/1.51	.50^**^
Understanding body language is as important as verbal communication in physician–patient relationships. (4)	**.641**	.260	−.178	−.231	6.00/1.09	.53^**^
A physician’s sense of humor contributes to a better clinical outcome. (5)	**.593**	.116	−.174	−.081	5.57/1.34	.47^**^
Patients feel better when their physicians understand their feelings. (2)	**.569**	.387	−.246	−.215	6.07/1.78	.55^**^
Asking patients about what is happening in their personal lives is not helpful in understanding their physical complaints. (12)	.215	**.771**	.124	.066	5.68/1.35	.65^**^
Patients’ illnesses can be cured only by medical or surgical treatment; therefore, physicians’ emotional ties with their patients do not have a significant influence in medical or surgical treatment. (11)	.133	**.714**	.061	.287	5.50/1.44	.60^**^
I believe that emotion has no place in the treatment of medical illness. (14)	.211	**.677**	.270	.105	5.66/1.34	.63^**^
I do not enjoy reading nonmedical literature or the arts. (19)	.034	**.673**	−.004	.008	5.74/1.49	.47^**^
Attention to patients’ emotions is not important in history taking. (7)	.258	**.628**	.160	.213	5.86/1.28	.64^**^
It is difficult for a physician to view things from patients’ perspectives. (3)	−.061	.173	**.835**	.025	3.86/1.41	.26^**^
Because people are different, it is difficult to see things from patients’ perspectives. (6)	−.013	.311	**.764**	−.062	4.21/1.50	.34^**^
Physicians’ understanding of their patients’ feelings and the feelings of their patients’ families does not influence medical or surgical treatment. (1)	−.085	.162	.046	**.777**	4.21/1.78	.27^**^
Attentiveness to patients’ personal experiences does not influence treatment outcomes. (8)	.072	.312	−.058	**.719**	4.92/1.68	.42^**^
Physicians should not allow themselves to be influenced by strong personal bonds between their patients and their family members. (18)	−.271	−.151	.261	.202	2.83/1.50	.-.08
Eigenvalue	5.20	3.12	1.64	1.46		
% Variance	26.01	15.59	8.21	7.28		

^a^Items are listed by the order of magnitude of the factor coefficients within each factor. Factor loadings equal to or greater than 0.4 are in bold. Numbers in parentheses represent the sequence of the items in the actual scale. Items were scored using a seven-point Likert-type scale

r_it_ = Item-total score correlation. ** Indicates statistical significance levels *p* < 0.01

### Factor structure

The results of the factor analysis are presented in Table 3. Four factors emerged. Factor 1 was a major component that can be labeled as “Perspective taking” with ten items loading above 0.50 and accounting for 26.01% of the variance. Factor 2 can be considered as “Compassionate care,” with five items loading above 0.50, accounting for 15.59% of the variance. Factor 3 accounted for 8.21% of the variance and can be considered as “Standing in the patient’s shoes,” with two items loading above 0.5. A fourth factor comprised items 1 and 8 and was uninterpretable. One item (item18) failed to load onto any of the factors in accordance with standards recommended by Tabachnick and Fidell [8].

### Gender differences in JSE-S scores

**Fig. 1:**
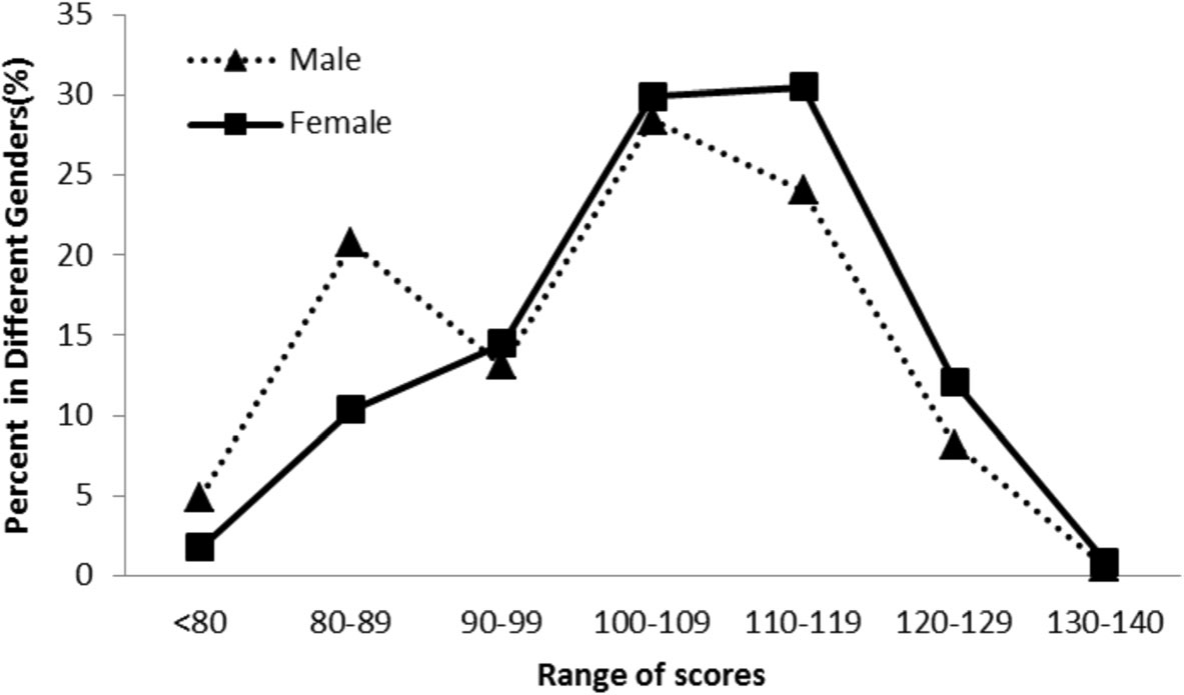
Scores distributions in different genders. The figure illustrates the distribution of empathy scores by gender. More than half of the male and female participants scored between 100 and 119. Taking the interval that includes the average score of 104.66 as the boundary, it can be seen that the proportion of men in the lower segment (less than 100) is higher than that of women, and the proportion of women in the higher segment (greater than 109) is higher than that of men. Overall, women scored higher than men, and the difference was statistically significant

The results examining group differences are reported in Table 4. With regard to gender differences, the mean empathy scores for female and male students were 106.06 (*SD* = 12.68) and 102.07 (*SD* = 13.46), respectively, and the *t*-test indicated that the difference was significant. Figure 1 shows the empathy score distributions for the different genders.

**Table 4:** Group comparisons of empathy scores on the JSE-S

Characterteristic	N(%)	Median	Mean ± SD	*P*-value
Global scores	521 (100.00%)	106.00	104.66 ± 13.09	
Gender**				0.001
Male	183 (35.12%)	104.00	102.07 ± 13.46	
Female	338 (64.88%)	107.00	106.06 ± 12.68	
Student cadre or not*				0.019
Yes	334 (64.11%)	106.00	105.66 ± 12.74	
No	187 (35.89%)	104.00	102.86 ± 13.54	
Future career preference**				0.003
Becoming a doctor^a^**	393 (75.43%)	106.00	105.32 ± 13.20	0.002
Not becoming a doctor	26 (4.99%)	99.00	96.23 ± 13.34	
Not yet decided^a^*	102 (19.58%)	104.00	104.25 ± 11.88	0.005
Father’seducational status*				0.030
High school or below	189 (36.28%)	108.00	106.30 ± 12.47	
Higher education	332 (63.72%)	105.00	103.72 ± 13.36	
Mother’s educational status				0.090
High school or below	219 (42.03%)	106.00	105.80 ± 12.77	
Higher education	302 (57.97%)	105.00	103.83 ± 13.28	

^a^compared to not becoming a doctor. *P < 0.05,**P < 0.01

### Group comparison by student cadre or not

In China, student organizations are spontaneously composed of students based on their interests, hobbies, projects, and so on. The student organization is constituted of several functional departments, and every department has its own affairs and responsibilities and has 3 to 10 student cadres. A student cadre is what may be considered a type of student leader, though it is much more than that. The results of the *t*-test showed a significant difference in group scores, indicating that student cadres had higher levels of empathy.

### Future career preference

A significant difference in empathy was found among the groups as it relates to future career preference. Students who did not want to become doctors were lower in empathy than those who wanted to become doctors (96.23 vs 105.32) and those who did not plan on becoming a doctor (96.23 vs 104.25).

### Parent education status

We divided parents’ level of education into two groups: those who had a high school education or below; and those who graduated from college or received a bachelor’s, master’s, or doctoral degree, whom we classified as parents with higher education. A significant difference was found only for fathers’ education. Students whose fathers had a high school education or below had slightly higher levels of empathy than fathers with higher education (106.30 vs 103.72). There was no significant difference between mothers, although the pattern was similar to that of fathers (105.80 vs 103.83).

## Discussion

The mean empathy score of the sample in the present study is similar to that found in Japan [5], China [7], Korea [9], India [10], and Iran [11] and relatively lower than the mean scores reported in some Western countries, such as Spain [12], America [13], and Germany [14]. Thus, Asian students tend to have similar levels of empathy but lower than do Western students (Additional file 1: Table S1 in Supplemental Data). The differences in empathy are possibly related to cultural and social differences. For example, in Asian countries, high school graduates are admitted directly into medical colleges by passing a highly competitive and heavily science-oriented selection system [5, 7, 10, 15]. Usually, only when students attach great importance to science subjects, such as mathematics, physics, and chemistry, can they outperform others to emerge at the top of their high school graduating class and enroll in medical colleges. Those requirements for medical students come at the expense of the arts and humanities, which may be one of the reasons for their lower levels of empathy. Furthermore, communication patterns that place less emphasis on nonverbal communication, as well as differences in medical education among Asian medical schools, may also explain why Asian medical students show lower levels of empathy than their Western counterparts [16].

The factor analysis resulted in a four-factor solution. Factors 1, 2, and 3 represented three dimensions of empathy: perspective taking, compassionate care, and standing in the patient’s shoes. These three factors have also emerged in previous studies, in China as well as in other countries [5, 7, 17], in which perspective taking has been described as a major dimension of empathy in patient care. The fourth factor was not found in any previous studies. According to Velicer and Fava [18], a minimum of three items per factor is required for a stable factor. As such, factor 4 may not be a stable factor. A sudden drop in the magnitude of the pre-rotated eigenvalues after extracting the other factors also suggests the instability of the factor. Additionally, the item “Physicians should not allow themselves to be influenced by strong personal bonds between patients and their family members” (item 18) did not load on any factor and was not endorsed. An insufficient factor loading and a low correlation for this item appeared in other Asian samples [11, 19] as well as in an Australian sample [20]. Therefore, item 18 may not be an item that represents empathy in patient care in medical students in China.

In this study, we found that women were more empathic than men, which concurs with studies in most other countries [5, 9, 16, 21], and in particular, with a study of students studying a five-year medical training program in northern China [19]. However, there was no gender difference in empathy in a previous study of Chinese medical students studying an eight-year program [7]. This inconsistency may be attributable to sample selection. The eight-year program is only approved in a few top universities in China, and some of its unique features, including the extreme competitiveness and high career goals and expectations of eight-year programs, may diminish any differences in empathy between men and women. However, in the present study, we included medical students studying both five-year and eight-year programs and three medical schools. In addition, because the sample size in the present study was larger than that of the previous one, and the difference in scores approached significance in the previous study, the smaller sample size might have accounted for not finding a significant difference.

Another noteworthy finding is that students who held positions as student cadres had higher empathy scores. Firstly, student cadres need to work together to resolve problems and accomplish tasks in their daily organizational activities. Thus, teamwork is their most common form of organizational activity. They must consider their teammates’ needs for belongingness and maintain an affiliation with each other, which requires them to recognize others’ emotions and concerns [22, 23]. Considering that the student cadres in an organization usually major in different specialties in the school, such altruistic inter-professional teamwork with a common value or purpose of serving other students is beneficial to the cultivation of empathy, based on previous research [24]. Secondly, student cadres usually spend more time engaged in community service activities, and previous research showed a trend in which the more hours students spent engaged in such activities, the higher their levels of empathy [25]. Most of these activities are medically related. For example, some student organizations at Fudan University provide students with the chance to be a volunteer for medical guidance at cooperative hospitals. In this process, student cadres try to adopt the patient’s perspective and gain a better understanding of the patient’s situation, concerns, and feelings. Consequently, students may develop higher sensitivity toward the patients, which could increase their empathic ability [26]. Additionally, volunteer experiences in clinical settings usually occur as soon as the student enrolls at the university, and a systematic review of the effect of early practical experience concluded that it fostered empathic attitudes toward patients [27]. Finally, student cadres act as a bridge between the school and the students; interacting with diverse groups of people including but not limited to students, teachers, and social groups. To enable them to communicate effectively with others, communication skills training is carried out regularly. Communication skills figure strongly in interventions aimed at enhancing empathy and are now used as an educational strategy to foster empathic ability in some medical schools [16]. Therefore, training in communication skills likely enhances empathy in student cadres. This finding is particularly informative because few teaching plans on empathy development have been established in Chinese medical schools, and it suggests that educators should consider student organizations as a potential modality through which empathy can be cultivated in medical students.

Even though China’s medical reform is entering a new period, tense patient-physician relationships have not been alleviated [28]. Some medical students are considering working in industries outside of the clinical setting as well as choosing careers totally unrelated to medicine [29]. Our findings showed that students who preferred not to become doctors had the lowest empathy scores, which were also lower than those of students who preferred other career options. This result is in accordance with that of the previously mentioned study of the eight-year medical program [7]. It is possible that poorly covered empathy cultivation in teaching plans, the prioritization of acquiring medical expertise over humanistic knowledge, and a conflict between students’ career goal and what is actually fulfilling to them in their training might lead to a decline in empathy and may account for this difference in empathy among the students.

Results from studies on the effect of parents’ education level on empathy in medical students are inconsistent. While some studies have found no significant relationship [30, 31], one study revealed that the mother’s education status affected medical students’ levels of empathy [32]. Conversely, we found the father’s education status to be associated with levels of empathy; students whose fathers’ education was high school or below were higher in empathy than those students whose fathers had attained a more advanced education.

This finding might be due to cultural and social differences. Following the reform and the open-door policy in China in the late 1970s, there have been enormous social and economic changes. These changes have, in turn, caused fundamental changes in a person’s position in the social hierarchy, which is basically determined according to occupation, education, capital, and income level [33]. Higher education generally leads to better occupations and a higher income; thus, physicians, lawyers, teachers, engineers, managers, administrators, and accountants have climbed the hierarchy [33], becoming the middle or upper class. Those with lower levels of education, such as employees in the service sector, workers, peasants, and the unemployed are more likely to receive a lower income and have shifted into the lower class. The burgeoning middle- and upper-class view education as a reliable means to achieve high social status, and therefore parents place great importance on their children’s academic achievements [34]. Ignoring humanistic values and promoting scientific ones is not likely to be conducive to the development of empathy in children.

Another possible explanation is related to the psychology of social class. Studies investigating the relationship between social class and emotion showed that people from lower socioeconomic classes were more empathic than those of a higher socioeconomic status [35, 36]. Additionally, the association between social class and empathic accuracy was investigated [36, 37], and people of lower-class status demonstrated greater empathic accuracy than people of the upper class. In light of these findings, using the father’s level of education as a proxy for socioeconomic status might explain the differences in participants’ levels of empathy.

Another consideration as to why the father’s education status may be related to empathy in medical students is the one-child policy in China. Parents, especially those who attained higher education, tend to have only one child. A significant correlation between the father’s higher education and having only one child was found in the present study (*r* = − 0.450, Additional file 1: Table S2 in Supplemental Data). There is evidence that the one-child policy has affected parenting [38, 39], such as inflicting increased pressure on the child to succeed in school [38]. Currently, in the Chinese society, people are often judged by what academic degrees they hold and from which universities. As they have only one child, parents tend to have a high degree of anxiety over their child having an advantage when competing for academic and job positions [40]. Pressure and anxiety exist both in the education system and in the family, which ultimately is passed on to the student. In a study including 2400 Chinese students of different ages in six cities and provinces, 76.4% of the students reported being in a bad mood because of pressure and high parental expectations, and some of them even reported feelings of despair [41]. Academic stress not only affects psychological health but extends to social relationships with peers and attitudes toward society at large. As a result of intense academic competition, feelings of jealousy, distrust, and animosity have been found to be common in peer relationships [40]. Such negative emotional states can impede the development of empathy [15, 42].

### Limitations

There are several limitations to this study. First, the three medical schools are all in Shanghai and therefore may not be representative of the national experience. Future research should include other university populations. Second, selection bias may have affected the findings. It is possible that students with higher empathy were more likely to complete the questionnaire online, and therefore we are unable to know the characteristics of non-responders. Finally, the JSE-S is a self-report instrument and susceptible to socially desirable responses, which can lead to response bias. Although a prior study showed that the social desirability response bias did not distort the validity of the scores [17], future studies are needed using instruments such as observer ratings and patient assessments to obtain more comprehensive and objective results.

## Conclusion

We believe that our findings not only provide important baseline data on empathy in Chinese medical students and reveal factors that affect their levels of empathy, but also enrich the available data on empathy in medical students around the world. The results suggest possible ways to improve the empathy level of medical students and call attention to the need to tailor interventions aimed at maintaining and fostering students’ empathy during medical school education, particularly within the context of medical education reform in many countries, including China.

## Supplementary information

**Additional file 1: Supplemental data Table S1.** Studies reporting the JSPE-S means for medical students. **Table S2.** Spearman rank correlation coefficients between the father’s educational status and only one child or not.

## Data Availability

Data presented in this manuscript have not been published elsewhere. Data from this project will not be shared. Consent was not sought from participants to share the data more widely than for the purposes of this study.

## Abbreviation

JSE-S: The Student Version of the Jefferson Scale of Empathy
