# Supplementary material for: A multi-institutional and cross-sectional study on empathy in Chinese medical students: differences by student cadre or not, future career preference, and father’s education status
Source: BMC Med Educ. 2020 Jan 28;20:24. doi: 10.1186/s12909-020-1935-x (PMC6988276; doi:10.1186/s12909-020-1935-x)
Supplement: Supplementary file 1 — Additional file 1: Supplemental data Table S1. Studies reporting the JSPE-S means for medical students. Table S2. Spearman rank correlation coefficients between the father’s educational status and only one child or not. [file 12909_2020_1935_MOESM1_ESM.docx]

**Supplemental data**

Table S1: Studies reporting the JSPE-S means for medical students

| Authors | Country | Mean | SD | N | P value |
| --- | --- | --- | --- | --- | --- |
| Kataoka U. et al.  (2009) | Japan | 104.3 | 13.1 | 400 | 0.6793 |
| Rahimi-Madiseh M. et al.  (2010) | Iran | 105.1 | 12.9 | 181 | 0.6959 |
| Wen D. et al.  (2013) | China | 109.60* | 12.09 | 753 | 0.0000 |
| Shashikumar R. et al.  (2014) | India | 102.91 | 19.217 | 448 | 0.1036 |
| Park KH. et al.  (2015) | Korea | 105.90 | 12.80 | 5343 | 0.1697 |
| Li D. et al.  (2018) | China | 104.2 | 13.6 | 442 | 0.5936 |
| Hojat M.  (2009) | USA | 115.0* | 10.0 | 685 | 0.0000 |
| Hegazi I and Wilson I  (2013) | Australia | 109.07* | 14.937 | 404 | 0.0000 |
| Preusche I and Wagner-Menghin M (2013) | Germany | 110.52* | 12.49 | 516 | 0.0000 |
| Leombruni P. et al.  (2014) | Italy | 108.71* | 10.60 | 257 | 0.0000 |
| Ferreira-Valente A . et al.  (2016) | Spain | 117.56* | 10.43 | 1104 | 0.0000 |

* The mean empathy scores were compared with present study (n = 521, Mean = 104.66, SD = 13.09) by t-test the p-value is significant at the 0.05 level.

Reference for table 2

1. Kataoka U, Hitomi, Koide S, Norio, Ochi S, Koji, Hojat S, Mohammadreza, Gonnella S, Joseph. 2009. Measurement of Empathy Among Japanese Medical Students: Psychometrics and Score Differences by Gender and Level of Medical Education. Academic Medicine. 84(9):1192-1197.
2. Rahimi-Madiseh M, Tavakol M, Dennick R, Nasiri J. 2010. Empathy in Iranian medical students: A preliminary psychometric analysis and differences by gender and year of medical school. Medical Teacher. 32(11):471-478.
3. Wen D, Ma X, Li H, Liu Z, Xian B, Liu Y. 2013. Empathy in Chinese medical students: psychometric characteristics and differences by gender and year of medical education. BMC Medical Education. 13(1).
4. Shashikumar R, Chaudhary R, Ryali VS, Bhat PS, Srivastava K, Prakash J, Basannar D. 2014. Cross sectional assessment of empathy among undergraduates from a medical college. Med J Armed Forces India. 70(2):179-185.
5. Park KH, Roh H, Suh DH, Hojat M. 2015. Empathy in Korean medical students: Findings from a nationwide survey. Medical Teacher. 37(10):943-948..
6. Li D, Xu H, Kang M, Ma S. 2018. Empathy in Chinese eight-year medical program students: differences by school year, educational stage, and future career preference. BMC Medical Education. 18(1):241.
7. Hojat M. 2007. Empathy In Patient Care: Antecedents, Development, Measurement, and Outcomes. New York: Springer.
8. Hegazi I, Wilson I. 2013. Maintaining empathy in medical school: it is possible. Med Teach. 35(12):1002-8.
9. Preusche I, Wagner-Menghin M. 2013. Rising to the challenge: cross-cultural adaptation and psychometric evaluation of the adapted German version of the Jefferson Scale of Physician Empathy for Students (JSPE-S). Advances in health sciences education: theory and practice. 18(4):573-587.
10. Leombruni P, Di Lillo M, Miniotti M, Picardi A, Alessandri G, Sica C, Zizzi F, Castelli L, Torta R. 2014. Measurement properties and confirmatory factor analysis of the Jefferson Scale of Empathy in Italian medical students. Perspect Med Educ. 3(6):419-30
11. Ferreira-Valente A, Costa P, Elorduy M, Virumbrales M, Costa MJ, Palés J. 2016. Psychometric properties of the Spanish version of the Jefferson Scale of Empathy: making sense of the total score through a second order confirmatory factor analysis. BMC medical education. 16(1):242.

Table S2. Spearman rank correlation coefficients between the father’s educational status and only one child or not

| Father’s educational status | N | *r* value | P value |
| --- | --- | --- | --- |
| Only one child or not | 521 | -.450 | .000 |
